# Recent Advances in the Synthesis and Application of Vacancy-Ordered Halide Double Perovskite Materials for Solar Cells: A Promising Alternative to Lead-Based Perovskites

**DOI:** 10.3390/ma16155275

**Published:** 2023-07-27

**Authors:** Santhosh Murugan, Eun-Cheol Lee

**Affiliations:** 1 Department of Nanoscience and Technology, Graduate School, Gachon University, Seongnam-si 13120, Republic of Korea; 2 Department of Physics, Gachon University, Seongnam-si 13120, Republic of Korea

**Keywords:** perovskite solar cell, vacancy-ordered halide double perovskites, solar energy

## Abstract

Lead-based halide perovskite materials are being developed as efficient light-absorbing materials for use in perovskite solar cells (PSCs). PSCs have shown remarkable progress in power conversion efficiency, increasing from 3.80% to more than 25% within a decade, showcasing their potential as a promising renewable energy technology. Although PSCs have many benefits, including a high light absorption coefficient, the ability to tune band gap, and a long charge diffusion length, the poor stability and the toxicity of lead represent a significant disadvantage for commercialization. To address this issue, research has focused on developing stable and nontoxic halide perovskites for use in solar cells. A potential substitute is halide double perovskites (HDPs), particularly vacancy-ordered HDPs, as they offer greater promise because they can be processed using a solution-based method. This review provides a structural analysis of HDPs, the various synthesis methods for vacancy-ordered HDPs, and their impact on material properties. Recent advances in vacancy-ordered HDPs are also discussed, including their role in active and transport layers of solar cells. Furthermore, valuable insights for developing high-performance vacancy-ordered HDP solar cells are reported from the detailed information presented in recent simulation studies. Finally, the potential of vacancy-ordered HDPs as a substitute for lead-based perovskites is outlined. Overall, the ability to tune optical and electronic properties and the high stability and nontoxicity of HDPs have positioned them as a promising candidate for use in photovoltaic applications.

## 1. Introduction

Conventional methods of producing power are not usually good ones. Burning coal, oil, and gas can have a detrimental effect on the environment. For instance, the production of electricity emits harmful gases such as carbon dioxide and carbon monoxide. Similarly, fossil fuel mining causes pollution and global warming, and excessive use of water resources can have adverse environmental effects. Therefore, other renewable energy sources should be considered to mitigate these problems. Solar energy is a viable alternative to fossil fuels as this generates clean, abundant power, and reduces carbon footprints and greenhouse gas emissions. Once installed, solar panels generate emission-free energy without any water requirements. Currently, crystalline silicon solar cells are the dominant technology, but because of their high production cost and material expense, new photovoltaic (PV) technology that combines high efficiency with low manufacturing costs is being explored. Perovskite materials with the formula ABX_3_, comprising a monovalent cation (A), a divalent cation (B), and a halide (X), have gained attention because of their excellent photovoltaic performance, low raw material cost, and ease of manufacturing. Perovskite solar cell (PSC) technology has taken the industry by surprise, owing to its remarkable boost in power conversion efficiency (PCE) from 3.8% to 26.08% [1,2,3,4].

The photovoltaic properties of organic–inorganic metal-halide perovskite materials are exceptional. These materials possess a long charge diffusion length [5,6,7], direct and adjustable band gap [8,9,10,11,12], minimal carrier recombination [13,14,15], high carrier mobilities [16,17], high molar extinction coefficient [18,19], and strong visible spectrum absorption [20,21]. Despite these outstanding properties, the usage of lead is currently a controversial topic because of ecological and environmental concerns [22,23]. The toxic nature of lead and the poor stability of PSCs, while exposed to oxygen and moisture, are significant barriers to potential commercialization [24,25]. Significant endeavors have been attempted to create stable and nontoxic halide perovskites for use in solar cells [26,27,28,29,30,31,32]. To eliminate the use of lead in metal-halide perovskite structures, one straightforward approach is to substitute this with another divalent cation such as germanium or tin. However, these elements have high-energy 5 s and 4 s orbitals, making them vulnerable to transitioning from their +2 state to a +4 state through oxidation [33,34,35]. This process causes the perovskite material to quickly degrade, which is damaging for long-term applications. To overcome these issues, The A_3_B(III)_2_X_9_ structure is produced by using the trivalent lone pairs of bismuth (Bi^3+^) and antimony (Sb^3+^) cations [36,37,38]. However, because of the low-dimensional structure, large band gap, defect intolerance, and high effective mass of carriers, the photovoltaic performance of the associated devices is poor.

One strategy for removing lead from the active layer of PSCs is to substitute two Pb^2+^ ions with two metal ions that have distinct characteristics, such as +1 and +3 oxidation states. By making this substitution, the perovskite crystal structure can be maintained while also having an active layer that is free of lead. These nontoxic cations are referred to as heterovalent metal cations. The subsequent material is called halide double perovskite (HDP) and has the formula A_2_B(I)B(III)X_6_ [39]. An alternative approach to maintaining charge neutrality in the crystal structure is to employ a single cation with a valence of four and a vacancy site to produce A_2_B(IV)X_6_. Tetravalent ion-containing double perovskites are regarded as analogs of A_2_B(I)B(III)X_6_ that are vacancy-ordered HDPs [40,41]. By selecting different elements for the A, B(I), B(III), B(IV), and X positions in the double perovskite structure, the existence of >100 stable double perovskites with appropriate structural and octahedral factors have been predicted [42]. The diverse options for these positions enable the creation of a range of stable double perovskites that may provide a promising option for various optoelectronic applications. The structural and functional diversity and excellent stability mean these perovskites could potentially serve as a lead-free substitute.

In the early 1960s, K_2_TiBr_6_, Rb_2_TiBr_6_, and Cs_2_TiBr_6_ were synthesized using fused SbBr_3_ as the solvent [43]. Cs_2_NaAmCl_6_ was later synthesized by drying an HCl solution containing cations [44]. Then, using the same technique, several HDPs (Cs_2_NaM(III)Cl_6_) were produced [45]. In 2016, Adam et al. synthesized Cs_2_AgBiBr_6_ and reported this as a possible substitute for the lead halide perovskites for its excellent photovoltaic properties [39]. In the same year, Eric et al. produced Cs_2_AgBiBr_6_ and Cs_2_AgBiCl_6_ from both solid-state and solution routes that exhibited light absorption at the visible range of the spectrum [46]. Afterward, many types of HDPs, including Cs_2_NaBiX_6_ [47], Cs_2_AgInX_6_ [48], Cs_2_AgSbX_6_ [49], (CH_3_NH_3_)_2_AgBiX_6_ [50], Cs_2_SnX_6_ [51], and Cs_2_TiX_6_ [52], were reported in the application of optoelectronics.

Vacancy-ordered HDPs have the potential for modifying the composition, structure, and dynamics at the A, B, and X sites for the desired optical and electronic properties. The band gap can be adjusted by selecting X ions and using strong covalent B–X interaction. Large halides allow low carrier effective masses and potentially higher mobilities. Tetravalent ions at the B-site and Cs^+^ at the A-site can achieve the desired properties of thermodynamic stability and resistance to humidity. These tunable optical and electronic properties and the high stability and nontoxicity of vacancy-ordered HDPs have positioned them as promising candidates for use in photovoltaic applications.

Initially, the compound Cs_2_SnI_6_ has been favorably utilized as a hole transport material (HTM) in solid-state dye-sensitized solar cells, achieving an efficiency of approximately 8% [51]. Similarly, Cs_2_TiI_2_Br_4_ and Cs_2_TiBr_6_ are deemed suitable materials for both single- and multi-junction perovskite solar cells due to their favorable band gap, improved environmental stability, and tolerance to defects. These promising compounds can attain a maximum efficiency of 3.3% [53]. In the field of vacancy-ordered HDP solar cells, a recent study revealed that Cs_2_PtI_6_ perovskite achieved the highest power conversion efficiency of 13.88% [54]. Presently, researchers are actively exploring different variations to further enhance the power conversion efficiency of A_2_BX_6_ perovskites.

Furthermore, the performance of PSCs utilizing vacancy-ordered HDP materials has been comparatively lower when compared to their lead-based counterparts, primarily due to inherent and extrinsic defects. Synthesizing lead-free vacancy-ordered HDPs is complex due to their quaternary nature, low precursor solubility, and high annealing temperature requirements. Obtaining high-quality HDPs in crystal or thin film form is challenging compared to ABX_3_ materials, with limited solubility of precursors being a major factor. Therefore, conducting a timely review to summarize the research advancements in this area is crucial to establish feasible strategies for optimizing the perovskite layer and facilitating discussions on recent breakthroughs in vacancy-ordered HDP-based materials. This review begins with a structural analysis of HDPs, followed by a summary of the synthesis methods for vacancy-ordered HDPs and their impact on material properties. The focus then shifts to recent advances in vacancy-ordered HDPs for solar cells that are classified according to their role in the device, including active and transport layers. Additionally, recent simulation studies are discussed. Finally, the review concludes by outlining the potential of this innovative family of materials as a substitute for Pb-based perovskites.

## 2. Structure and Formability of HDPs

**Figure 1:**
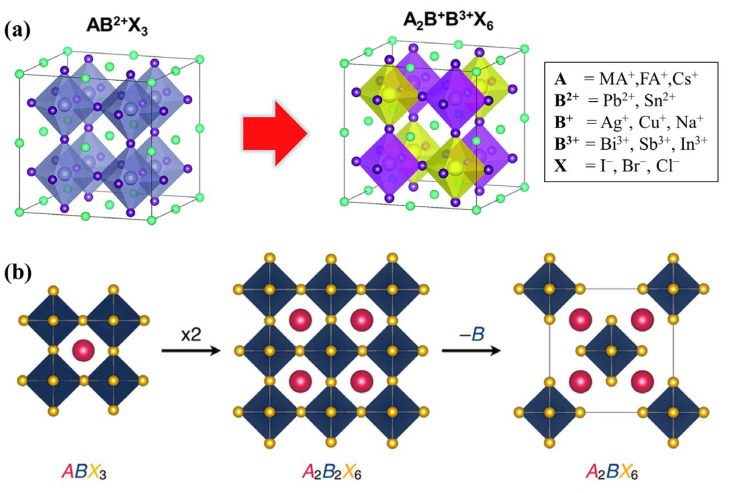
(**a**) Diagram of substitution B position cations in halide perovskites (AB(II)X_3_) with HDPs (A_2_B(I)B(III)X_6_). Reproduced by permission from [57], Copyright 2018 Royal Society of Chemistry. (**b**) The connection between the standard perovskite structure (ABX_3_) and the vacancy-ordered HDP structure (A_2_BX_6_). Reproduced by permission from [40], Copyright 2019 American Chemical Society.

CaTiO_3_ serves as the source of the ABX_3_ formula that defines the perovskite crystal structure [55]. The crystal structure of halide perovskites is recognized as being critical to their electronic characteristics, and the high Oh symmetry of typical lead-based halide perovskites contributes significantly to their outstanding electronic properties [56]. The ideal HDP crystal structure with the general formula A_2_B_2_X_6_ is developed from the conventional cubic perovskite archetype ABX_3_ through cation transformation (Figure 1a) [57]. Consequently, the HDP crystal structure has several characteristics with those of conventional halide single perovskites. HDPs usually contain two nontoxic B-cations that are split in ABX_3_, such as B(I)X_6_ and B(III)X_6_. This occurs when the cuboctahedral cavity center is occupied by the same A-site cation, such as CH_3_NH_3_^+^ (MA^+^) or Cs^+^ [39], which creates a network structure of alternating B(I)X_6_ and B(III)X_6_ octahedrons at the shared corners, known as rock-salt ordering [58]. This crystal structure enables the substitution of halogens at the X-site, of metal cations in various oxidation states at the B-site, and of inorganic and organic compounds at the A-site.

As shown in Figure 1b, the HDP A_2_B_2_X_6_ adopts a vacancy-ordered structure when one B-site cation is absent. The structure of A_2_BX_6_ shares many similarities with cubic ABX_3_ perovskites. The ability to modify the composition of all three sites (A, B, and X) in vacancy-ordered HDPs offers a vast phase space with significant opportunities for adjusting their structural, compositional, and dynamic properties [40]. This tunability enables the achievement of desirable electronic and optical properties. For example, the selection of X ions and the strong covalent interaction between B and X-site can modify the band gap of vacancy-ordered HDPs [59]. The close-packed lattice formed by the large halides facilitates significant orbital overlap between adjacent isolated octahedra. This interaction leads to lower effective masses of carriers and the potential for higher carrier mobilities. The structure can accommodate a wide range of tetravalent ions at the B-site to achieve the desired properties. In vacancy-ordered HDPs, the choice of the B-site can significantly impact the defect chemistry and charge transport characteristics. The substitution of Cs^+^ for MA^+^ or CH(NH_2_)_2_^+^ (FA^+^) in all-inorganic cesium halide perovskites has rendered them hopeful contenders owing to their remarkable thermodynamic stability and resistance to moisture [60,61].

The Goldschmidt tolerance factor (*t*) and the octahedral factor (μ) are two characteristics that are often considered when determining the crystalline stability of perovskites [62,63]. The tolerance factor, a dimensionless quantity, plays a crucial role in quantifying the stability and distortion of crystal structures. It becomes particularly important when selecting specific combinations of cations for a material. The stability of BX_6_ octahedra can be predicted using μ, which is the ratio of the ionic radius of the cation at the B-site (*r_B_*) to that of the anion at the X-site (*r_X_*). The X_6_ octahedron forms the octahedral cavity, which limits the size of the B-site cation.

(1) $$t=\frac{r_{A}+r_{B}}{\sqrt{2}\;\left(r_{B}+r_{X}\right)},$$

(2) $$\mu =\frac{r_{B}}{r_{X}},$$

where *r_A_* is the ionic radius of A-site cation. For HDPs, *r_B_* is considered as the average radius of the B(I) and B(III) cations.

The degree of symmetry falls when the tolerance factor deviates from 1.0. The value of t must be between 0.81 and 1.11 to form a stable perovskite structure while the octahedral factor should be in the range of 0.44–0.90. To enhance the accuracy of determining the structure of vacancy-ordered HDPs, it may be necessary to refine and adjust the analytical parameters for improved precision. The stability of vacancy-ordered HDPs can be ascertained by considering the ratio of the radius of the A-site cation to that of a 12-coordinate void, using the tolerance and octahedral factors as references [41,64]. During cooling, the vacancy-ordered structure undergoes a phase transition that reduces the symmetry, which is attributed to the cooperative tilting and rotations of the octahedra in the crystal structure. A discrepancy in the ionic radii of the component atoms can affect these phase transitions.

However, according to previous studies, the ability of the tolerance factor to accurately differentiate between perovskite and non-perovskite materials is limited. It was found to be effective in only 74% of the 576 types of ABX_3_ solids that were experimentally examined under ambient conditions. Furthermore, the accuracy of the tolerance factor was significantly reduced for materials that included halides with an atomic weight higher than that of fluorine or oxygen, indicating that the method may be less effective for specific types of compounds [65]. The possibility of using the tolerance factor for the discovery of new materials is greatly constrained by this lack of generality to halide perovskites. A factor termed atomic packing fraction (η) was developed by Sun et al. and was used to predict the thermodynamic stability of double perovskites with an accuracy of 86% [66]. Bartel et al. recently derived a new tolerance factor (*τ*) that produced an accuracy >90% for halide-based perovskites [65].

(3) $$\tau =\frac{r_{X}}{r_{B}}-n_{A}(n_{A}-(\frac{\frac{r_{A}}{r_{B}}}{\ln\left(\frac{r_{A}}{r_{B}}\right)})).$$

The oxidation state of *A* is represented by *n_A_*. This discovery enables a completely new physical understanding of stability. It also allows for making predictions for novel stable single and double inorganic and organic–inorganic hybrid perovskites. The stability of perovskite is directly linked to *τ*, where a decrease in *τ* consistently produces a higher probability of the material being classified as a perovskite. By contrast, the structural stability does not consistently increase or decrease with changes in the Goldschidmt tolerance factor *t*. Empirical evidence suggests that when *τ* is <4.18, stable perovskites are expected to form. Furthermore, instead of the typical double-variable descriptor (*t*, μ), *τ* can be used together with μ, as a single-variable descriptor, producing a more insightful index for predicting the structural stability of new stable inorganic and hybrid single and double perovskites. A recent study predicted the stability of HDP crystal structures using machine learning approaches combined with calculations based on first-principles density functional theory (DFT) [67]. The predicted stabilities of the method are consistent with the available experimental data.

In 1964, Brown postulated that whether a particular A_2_BX_6_ structure forms in a vacancy-ordered double perovskite is influenced by the ratio of the cavity size to the ionic radius of the A-site, similar to how the tolerance factor dictates the formation of ABX_3_ structures [64]. Karim et al. modified Brown’s equation and presented a new radius ratio (R) for A_2_BX_6_ compounds [68]. Brown defined that the distance between the centers of the A-site ion and the surrounding halide ions d(AX) is determined by the coordination sphere of the surrounding halide ions.

(4) $$d\left(AX\right)=\frac{d\left(X_{\alpha }X_{\beta }\right)+d(X_{\alpha }X\alpha )}{2},$$

(5) $$R=\frac{r_{A}+r_{B}}{\frac{1}{2}\left[d\left(X_{\alpha }X_{\beta }\right)+d(X_{\alpha }X_{\alpha })\right]},$$

where d(X_α_X_β_) and d(X_α_X_α_) are the interhalogen distances. The cation at the A-site cannot fit into the cavity if R is >1. If d(X_α_X_α_) = d(X_α_X_β_), Equation (5) can be simplified to the widely recognized Goldschmidt tolerance factor equation. Thus, the Goldschmidt tolerance factor equation is a particular instance of the broader Equation (5).

## 3. Crystal Synthesis and Film Preparation of Vacancy-Ordered HDP

The complexity of synthesizing lead-free vacancy-ordered HDPs becomes evident upon entering this field and is caused by the quaternary nature of these compounds, the low solubility of precursors, and the requirement for high annealing temperatures [69,70,71]. The presence of these factors complicates the process of obtaining high-quality HDPs that are phase pure and that are categorized based on their physical state, such as crystals or thin films, when compared with the more commonly used ABX_3_ materials. In contrast to ABX_3_ perovskites, a considerable focus in the synthetic methods of vacancy-ordered HDPs is placed on crystal synthesis rather than thin-film production. This emphasis is partly attributed to the complex nature of the material and the limited solubility of precursors.

### 3.1. Crystal Synthesis

Crystal synthesis is a crucial initial step for several methods of producing thin films. In this context, this discussion briefly covers crystal synthesis involving various types of crystals such as macroscopic single crystals, polycrystalline powders, and nanocrystals (NCs). Solution-processing techniques are the most common method for synthesizing vacancy-ordered HDPs and involve using a chemical precursor solution where one or more elements of the final compound are dissolved or dispersed in a solvent. This method can be applied to a broad range of compositions within the perovskite-derived material class. However, in this discussion, we will limit our focus to examples of A_2_BX_6_ compounds. 

#### 3.1.1. Solution-Based Processing

**Figure 2:**
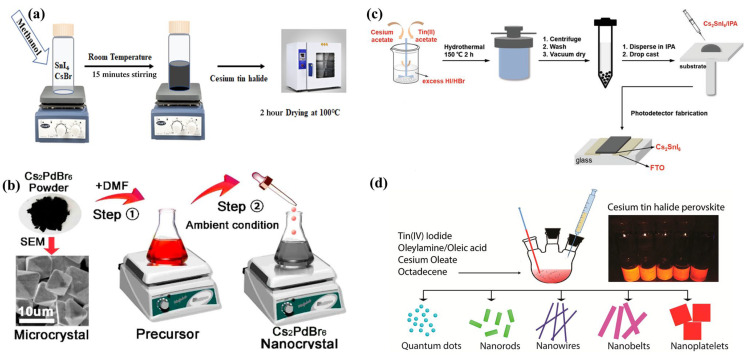
Schematic of the procedure involved in synthesizing (**a**) Cs_2_TiBr_6_ perovskite via a straightforward one-step solution process. Reproduced by permission from [73], Copyright 2022 John Wiley and Sons. (**b**) Cs_2_PdBr_6_ NC via an antisolvent route. Reproduced by permission from [75], Copyright 2018 American Chemical Society. (**c**) Cs_2_SnI_6_ from the hydrothermal method. Reproduced by permission from [76], Copyright 2019 John Wiley and Sons. (**d**) Diverse morphology Cs_2_SnI_6_ NCs by hot-injection method. Reproduced by permission from [79], Copyright 2016 American Chemical Society.

In 2014, Lee et al. introduced Cs_2_SnI_6_ polycrystals synthesized through a simple and efficient solution-phase method as a promising material for use in next-generation solar cell technology [51]. A mixture of aqueous HI and Cs_2_CO_3_ was used to create a concentrated acidic solution of CsI while SnI_4_ was dissolved in warm absolute ethanol to create an orange solution. A fine black powder was obtained by vigorously stirring the two solutions, which were subsequently combined. The mixture was filtered and then rinsed with pure ethanol, producing a complete yield. Black truncated octahedral crystals of Cs_2_SnI_6_ were generated by the introduction of a SnI_4_ solution with a low alcohol concentration into an aqueous solution of CsI via liquid diffusion. The stability of this compound was maintained by its ability to resist air and moisture even at room temperature. Qiu et al. modified Lee’s method to produce needle-shaped Cs_2_SnI_6_ crystals [72], where equimolar amounts of CsI and SnI_2_ were sequentially added into a mixture of aqueous HI and H_3_PO_2_ while heating to 120 °C. Yellow needle-shaped crystals precipitated inside the precursor, and black-colored Cs_2_SnI_6_ powders with a 1.48 eV band gap were obtained after drying. Qamar et al. recently used the less hazardous solvent methanol in a straightforward one-step solution process, illustrated in Figure 2a, to produce a high-quality Cs_2_SnI_6-x_Br_x_ material [73]. Maughen et al. also modified Lee’s method for the preparation of Cs_2_Sn_1−x_Te_x_I_6_ solid solution, which involved using a precursor of SnI_4_/TeI_4_ [41].

Sakai et al. reported the synthesis and properties of a Cs_2_PdBr_6_ NCs created using a unique solution process via in situ oxidization of Pd^2+^ to Pd^4+^ [74]. Black Cs_2_PdBr_6_ single crystals were synthesized by dissolving 2 M CsBr and 1 M PdBr in aqueous HBr at 85 °C, followed by the addition of 10% dimethyl sulfoxide (DMSO) and cooling to room temperature. The crystals were washed with toluene and water, then dried on a hot plate and in a box oven. The subsequent material had a cubic crystal structure with high stability and a band gap of 1.6 eV, making it potentially useful for various optoelectronic applications.

Zhou et al. synthesized lead-free all-inorganic Cs_2_PdBr_6_ perovskite NCs with an average diameter of 2.8 ± 1.1 nm through a simple antisolvent method at room temperature [75]. Cs_2_PdBr_6_ microcrystals and NCs were synthesized through a modified procedure (Figure 2b). CsBr and PdBr_2_ were dissolved in HBr and heated to 120 °C, then DMSO was added to precipitate Cs_2_PdBr_6_ microcrystals. To obtain Cs_2_PdBr_6_ NCs, the microcrystals were dissolved in dimethyl formamide (DMF) and rapidly injected into propanoic acid to form a colloidal solution that was dried to obtain NC powders without further purification. This synthetic procedure is compatibility with various organic solvents, opening new possibilities for the application of these NCs in various fields. The calculated optical band gap for Cs_2_PdBr_6_ and Cs_2_PdI_6_ NCs was 1.69 and 1.41 eV, respectively, indicating a narrow band gap for both materials.

Han et al. introduced a novel and noncomplex solution-phase technique, known as the hydrothermal method, for synthesizing high-quality and high-yield Cs_2_SnX_6_ crystals (where X = Br, I) [76]. Synthesis of Cs_2_SnI_6_ or Cs_2_SnBr_6_ involved dissolving 0.004 mol cesium acetate and 0.002 mol tin (II) acetate in either 10 mL of hydriodic acid or 10 mL of hydrobromic acid, respectively. The mixture was stirred and then transferred to an autoclave for hydrothermal growth at 150 °C for 2 h (Figure 2c). The subsequent powders were washed with isopropanol and dried at 60 °C overnight for an approximate 91% yield for Cs_2_SnI_6_ and 80% for Cs_2_SnBr_6_. These perovskites exhibit a vacancy-ordered defect-variant structure, with half of the Sn sites being vacant while tin displayed a 4+ oxidation state. Notably, the band gaps for Cs_2_SnI_6_ and Cs_2_SnBr_6_ were 1.84 and 1.42 eV, respectively, indicating that both materials are highly promising for use in photovoltaic applications. Zhang et al. employed a similar method to synthesize zero-dimensional Rb_2_ZrCl_6_ microcrystals for use in light-emitting diodes [77]. The absorption spectrum of Rb_2_ZrCl_6_ was analyzed using a Tauc plot, which revealed an experimentally measured band gap of 4.97 eV.

The conventional hydrothermal method can be both time-consuming and demanding in terms of high temperature and pressure. To address these concerns, Cao et al. developed an innovative approach using ionic liquids (ILs) as a precursor solvent for the manufacture of vacancy-ordered HDPs [78]. This method produced higher-quality crystals than those prepared using the traditional hydrochloric acid system. ILs are ionic compounds that have a melting point < 100 °C and are commonly referred to as low-temperature molten salts. ILs are a useful substitute for conventional organic solvents because they are odorless and nonflammable and have a low vapor pressure and are, therefore, challenging to evaporate. ILs were used to prepare Cs_2_ZrCl_6_ and Cs_2_SnCl_6_ doped with Sb or Bi. ZrCl_4_ or SnCl_2,_ as well as BiCl_3_ or SbCl_3_, was added to a pretreatment liquid of 1-butyl-3-methylimidazolium chloride, stirred, and CsCl and hydrochloric acid was added. The precipitate was collected and washed with methanol and dried at 80 °C for 3 h. The study revealed that Cs_2_SnI_6_ and Cs_2_SnBr_6_ have band gaps of 1.84 and 1.42 eV, respectively, indicating that both materials have potential for use in optoelectronic applications.

In a simple and affordable phosphine-free hot-injection method, Wang et al. achieved the first-ever synthesis of Cs_2_SnI_6_ NCs with diverse morphologies such as spherical quantum dots, nanorods, nanowires, nanobelts, and nanoplatelets (Figure 2d) [79]. The commercial precursors employed were inexpensive and non-toxic, making the subsequent Cs_2_SnI_6_ NCs ideal for the development of earth-abundant, less toxic, and environmentally friendly optoelectronic devices that can be manufactured through solution processing at a low cost. The use of inexpensive and nontoxic commercial precursors in the production of Cs_2_SnI_6_ NCs makes them an excellent candidate for the development of low-cost, environmental-friendly optoelectronic devices that use abundant earth resources and have lower toxicity levels. Moreover, the solution-processing manufacturer further enhances their viability for practical applications. The synthesis of Cs_2_SnI_6_ NCs involved loading octadecene and SnI_4_ into a three-neck flask with degassing under vacuum. A mixture of oleylamine and oleic acid was heated and then the Cs-oleate solution was injected with vigorous stirring, forming the NCs. The reaction mixture was cooled and purified with toluene and hexane. The synthesis and purification processes did not require an argon-filled glovebox. Grandhi et al. adapted Wang’s method and conducted the first synthesis of Cs_2_TiBr_6_ NCs [52]. These were synthesized at a temperature of 185 °C and had a band gap of 1.9 eV and demonstrated a considerable level of stability for eight weeks when suspended. By adjusting the temperature of the hot injection, the size and morphology of the synthesized Cs_2_TiBr_6_ NCs could be controlled. Recently, Jin et al. described a method for synthesizing Bi^3+^-doped Cs_2_SnCl_6_ NCs with controlled properties through hot injection using HCl as the source of chloride [80].

#### 3.1.2. Solid-State Route

A different method for synthesizing Cs_2_TiBr_6_ perovskite powder involves using melt-crystallization. This technique requires a melting medium and uses high heating temperatures, without the need for additional solvents to successfully synthesize various metal-halide perovskite films. Ju et al. used melt-crystallization to produce Cs_2_TiI_x_Br_6−_ HP powders [81]. First, a specific amount of CsI, CsBr, TiI_4_, and TiBr_4_ was added to quartz tubes that were then vacuumed to a pressure of approximately 10^−6^ Torr and sealed using an oxy-methane torch. Subsequently, the sealed tubes were heated at a rate of 10 °C.min^−1^ to 700 °C and held at this temperature for 72 h. Afterward, they were cooled gradually at 10 °C.min^−1^ until room temperature. Finally, the tubes were opened within a glovebox containing nitrogen gas to remove the powder form of Ti-based vacancy-ordered HDPs. The band gaps of roughly 1.38 and 1.78 eV were observed in Cs_2_TiI_2_Br_4_ and Cs_2_TiBr_6_ perovskites, respectively, indicating their suitability for use in single-junction PSCs and tandem PV applications.

Mechanochemical ball milling is a synthesis technique that repeatedly deforms and reduces the size of precursor materials within a sealed crucible. As the jar and disk center rotate together, the milling balls detach from the crucible wall and impact other balls or precursor powders, causing deformation and rupture that reduces particle size, increases surface area, and homogeneously mixes the materials. The resulting powder from this method can be readily extracted and sieved, providing advantages over other synthesis techniques. These advantages include easy scalability and independence from precursor solubility, allowing for a broader range of reactants to be utilized. This method is considered a “green” process as it does not require solvents, thereby eliminating the risk of incomplete solvent removal. However, contamination from the crucible or milling balls and an increase in crystal defect density due to high mechanical forces are potential limitations. Cho et al. used a mechanochemical reaction to synthesize perovskite powders of A_2_SnI_6_ (where A = Rb^+^, Cs^+^, MA^+^, or FA^+^) at ambient temperature via a ball-milling method [82]. The synthesis involved mixing AI and SnI_4_ with diethyl ether and ZrO_2_ balls and adding HI as an additive. Diethyl ether was used as a liquid medium to prevent powder agglomeration. The resultant slurry was dried at 60 °C for 6 h after ball milling for 18 h at 100 rpm. The subsequent Rb_2_SnI_6_ displayed a tetragonal structure due to significant octahedral tilting, whereas Cs_2_SnI_6_, MA_2_SnI_6_, and FA_2_SnI_6_ exhibited cubic structures. Incorporating HI while ball milling reduced the formation of secondary phases and changed the physical characteristics of the subsequent composite material. Kupfer et al. used a comparable approach to obtain phase-pure Cs_2_TiBr_6_, as well as its less-studied iodine-based counterparts, namely Cs_2_TiBr_4_I_2_, Cs_2_TiBr_2_I_4_, and Cs_2_TiI_6_ [83]. The Cs_2_TiBr_6−x_I_x_ family of materials displays indirect semiconductor behavior, and their respective band gaps are 1.88, 1.13, 1.04, and 1.02 eV for x values of 0, 2, 4, and 6, respectively. The direct band gap for these materials is approximately 0.1 eV larger than their corresponding indirect band gap.

Kaltzoglou et al. synthesized mixed-halide vacancy-ordered perovskite Cs_2_SnI_3_Br_3_ in two steps [84]. Initially, Cs_2_SnBr_6_ and Cs_2_SnI_6_ were obtained through separate processes, and subsequently, Cs_2_SnI_3_Br_3_ was synthesized using the solid-state synthesis method. The preparation of Cs_2_SnBr_6_ involved a reaction between SnBr_4_ and CsBr in DMF at a temperature of 100 °C. Cs_2_SnI_6_ was prepared by fusing SnI_4_ and CsI in a vacuum-sealed fused silica tube at 400 °C for 5 h. Finally, equimolar amounts of Cs_2_SnBr_6_ and Cs_2_SnI_6_ were reacted under vacuum at 400 °C for 5 h to produce Cs_2_SnI_3_Br_3_.

### 3.2. Thin Film Synthesis

The preparation of HDP thin films has been the focus of much research, and the two most widely adopted methods for their synthesis are vapor and solution deposition. These methods can successfully produce high-quality thin films that are necessary for the efficient operation of solar cells.

#### 3.2.1. Solution Processing

Chemical solution deposition is a cost-effective method that has gained extensive usage in fabricating ABX_3_ thin films and can be applied to HDPs. This is an attractive alternative to more expensive and complex methods, such as vapor deposition, and is particularly advantageous because this allows for large-area scale-up, which can be achieved by printing precursor inks. This scalability is particularly useful for large-scale manufacturing and commercial production of thin films. Another significant advantage is the facile control of the final stoichiometry and composition of the thin film. This is possible by directly adding the desired elements to the precursor solution, which allows for compositional alloying. By controlling the composition of the precursor solution, the properties of the thin film can be precisely tailored to the desired application. Nonetheless, the efficacy of the technique efficacy is limited by the solubility and purity of the precursor, as well as the incomplete elimination of organic and other residues.

Lee et al. developed a two-step coating process for applying Cs_2_SnI_6_ layers of different thicknesses [51]. In the first step, a CsI solution was prepared and applied onto a Fluorine-doped tin oxide (FTO) substrate using an electrostatic-assisted spray (E-spray) technique. The thickness of the CsI layer was regulated by modifying the spinning rate and electric field, and then the film was annealed at 130 °C for 10 min. In the next step, an ethanol solution of SnI_4_ was added dropwise onto the CsI layer until this turned black, signifying the successful synthesis of the Cs_2_SnI_6_ phase. Then, the black film was heated in air at 110 °C for 5 min. The drop casting was repeatedly performed until the CsI layer transformed into a black color. The band gap of the Cs_2_SnI_6_ material was approximately 1.3 eV when measured at room temperature. The material shows a low electrical resistivity of around 100 Ω·cm and behaves as an n-type semiconductor. Dolzhnikov et al. reported a simple method of drop casting for depositing Cs_2_SnI_6_ NCs into thin films that contain no surface ligands [85] and no apparent spaces or breaks at the interface between the film and the substrate. This method eliminates the need to remove organic material after synthesis, which is beneficial in optimizing electronic coupling between particles and minimizing contact resistance at the NC-electrode interface. Recently, Shanmugam et al. used the drop-casting method to fabricate a vacancy-ordered HDP thin film based on osmium [86]. A drop-casting Cs_2_OsX_6_ (X = Cl^−^, Br^−^, I^−^) solution was applied onto a cleaned FTO surface. The solution was made by combining 15 mg of Cs_2_OsX_6_ with 200 µL of isopropyl alcohol, 190 µL of Millipore water, and 10 µL of Nafion (a 5% *w*/*w* solution in water and 1-propanol) and ultrasonicated for 1 min. A 100 µL portion of the solution was drop-casted onto the FTO surface to create an active area of 0.5 × 0.5 cm^2^ by scraping out the excess film. The coated surface was then left to dry under ambient conditions. Attempts were made to deposit the material in steps, first by coating CsX followed by the metal precursor, and alternatively, by coating the metal precursor followed by CsX. However, this approach did not produce any perovskite material on the substrate.

**Figure 3:**
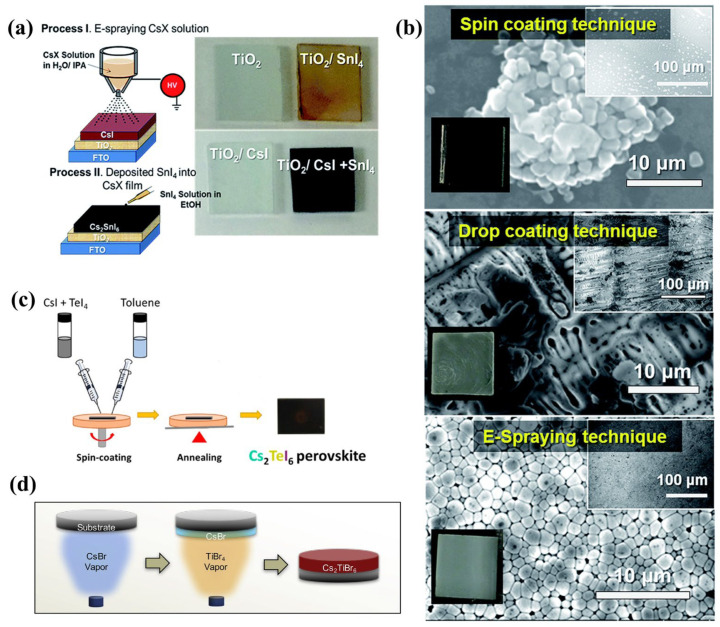
(**a**) Schematic of two-step deposition process for Cs_2_SnI_6_ films that involve E-spraying followed by drop coating; photo images of each process are shown. (**b**) Scanning electron microscopy images of CsI films were created through various methods including spin-coating deposition (1500 rpm, 30 s), dropping deposition, and E-spray deposition. Reproduced by permission from [89], Copyright 2019 Royal Society of Chemistry. (**c**) Spin-coating process of Cs_2_TeI_6_ films. (**d**) Vapor-based synthesis method of Cs_2_TiBr_6_ thin film. Reproduced by permission from [53], Copyright 2018 Elsevier.

Kapil et al. used a single-step spray-coating solution process to produce Cs_2_SnI_6_ thin films [87]. The first step involved preparing a solution with 300 mg/mL Cs_2_SnI_6_ in DMF by stirring the mixture at room temperature for 3 h. The use of dehydrated DMF was optimal for the solution preparation as even a small amount of water led to poor solubility of Cs_2_SnI_6_ and the formation of white precipitates due to decomposition. After the coating process, the films were heated at 130 °C for 5 min to remove the solvent. The rapid black coloration of the films within 1 min of heating indicated a swift crystallization process. By performing Hall measurements, a high mobility of 3.82 × 10^2^ cm^2^/ (V.s) was estimated for Cs_2_SnI_6_, which exhibits p-type characteristics. Xu et al. used an E-spray deposition technique to cultivate a thick Cs_2_TeI_6_ film in ambient conditions [88]. A solution of Cs_2_TeI_6_ with varying concentrations was prepared using a mixed solvent of DMF and ethanol with hydriodic acid. The solution was then coated onto a substrate using an E-spraying technique with a controlled spraying rate and electric field, where the duration of the E-spraying process controlled the thickness of the subsequent Cs_2_TeI_6_ film. Lee et al. developed a novel approach for coating Cs_2_SnI_6_ layers, using a two-step method that combines both E-spraying and drop casting [89]. Figure 3a illustrates the process where a solution containing CsI or CsBr powder in a solvent mixture of deionized water and isopropanol is electrosprayed onto TiO_2_ spheres. The duration of E-spraying determines the thickness of the coating, which is subsequently heated in a box furnace. In the second step, a solution of tin(IV) iodide or tin(IV) bromide in ethanol is drop-coated onto the CsI or CsBr layer until the color of the coating changes from black to light yellow, after which the film is heated in air. Scanning electron microscopy was used to explore how various coating methods affected the formation of CsI crystals (Figure 3b) and showed that spin coating produced cluster formation, drop coating produced dendritic crystallites, and E-spraying produced a thin film of crystallites.

Vazquez-Fernandez and colleagues reported that Cs_2_TeI_6_ thin films were initially fabricated using a spin-coating technique (Figure 3c) that produced a highly uniform film [90]. The study demonstrated that the spin-coating technique deposited stoichiometric quantities of CsI and TeI_4_ with a ratio of 2:1 to produce Cs_2_TeI_6_ films. The Cs_2_TeI_6_ films were synthesized on glass substrates using a controlled nitrogen atmosphere and solvents such as DMSO and DMF. The optimum process involved mixing CsI and TeI_4_ in DMSO (53 wt %) at a 2:1 molar ratio with dynamic spin coating at 2000 rpm for 15 s, and heating to 100 °C, which was maintained for 10 min for annealing. The addition of toluene as an antisolvent during spin-coating from DMSO, followed by annealing, produced better quality films than those produced using the one-step spin method without the antisolvent. The films were stable up to 250 °C and exhibited an optical band gap of approximately 1.5 eV, with absorption coefficients reaching roughly 6 × 10^4^ cm^−1^. Additionally, the films exhibited carrier lifetimes of approximately 2.6 ns for an unpassivated 200 nm film, a work function of 4.95 eV, and a surface conductivity that was p-type. Recently, Liu et al. used a solution-based spin-coating method to enhance the film quality of Cs_2_SnI_6_ perovskite by incorporating a small excess of SnI_4_ in a precursor solution containing DMF and DMSO [91]. The technique facilitated one-step solution processing, producing Cs_2_SnI_6_ thin films with high electron mobility, uniform morphology, and high crystallinity. Precursor solutions of Cs_2_SnI_6_ were prepared by dissolving CsI and SnI_4_ powders at various molar ratios in a mixed solvent of DMSO/DMF, and then films were fabricated using one-step spin coating of the precursor solution followed by low-temperature annealing at 100 °C for 5 min in a nitrogen-filled glove box. The authors highlighted the crucial influence of precursor engineering and Mn^2+^ doping in the modulation of both film quality and electronic properties of Cs_2_SnI_6_ perovskite films.

#### 3.2.2. Vapor-Based Deposition

Vapor deposition is a widely used technique in the field of materials science to produce thin films and offers a high degree of control over their composition, structure, and properties. This method involves the deposition of a thin layer of material onto a substrate surface through the vapor phase. Vapor deposition has been used for the fabrication of thin-film semiconductors and has also shown promising results in the production of ABX_3_ perovskites. This method could surpass the restrictions of solution deposition, including insufficient precursor solubility and the difficulty of achieving desired film thickness.

Saparov et al. reported a vapor deposition approach for producing thin films of Cs_2_SnI_6_ with superior quality [92]. Cs_2_SnI_6_ films were produced by evaporating CsI and these were subsequently annealed using SnI_4_ vapor in a nitrogen-filled glovebox. The films were deposited onto three different types of substrates, including glass, FTO, and FTO with a compact TiO_2_ layer. The fabrication process was precisely controlled to obtain a single-phase Cs_2_SnI_6_. The most favorable results were achieved when CsI films were annealed at 190 °C for 20–40 min in a preheated atmosphere of SnI_4_ for 10–30 min, then cooled slowly to 140 °C within approximately 10 min, and finally rapidly cooled. At higher annealing temperatures, impurities were observed, and above 250 °C, Cs_2_SnI_6_ could not be formed. As observed in earlier experiments, CH_3_NH_3_PbI_3_ films were less stable in air than stable Cs_2_SnI_6_ films having a 1.6 eV band gap. Qiu et al. made an exciting discovery that a film of B-γ-CsSnI_3_ undergoes a spontaneous transformation into an air-stable Cs_2_SnI_6_ film at room temperature when exposed to air [93]. By employing a two-step technique that integrated solid-state reaction and vapor deposition, a slender layer of B-γ-CsSnI_3_ was created on glass. The process involved cleaning the glass substrates with a mixture of solvents, followed by the deposition of CsI and SnI_2_ layers of equal thickness using thermal evaporation. Next, the layers were quickly annealed in a nitrogen environment to finalize the solid-state reaction. The subsequent B-γ-CsSnI_3_ thin films were black in color and air-stable. Upon exposure to air, the thin film underwent a phase change and spontaneously converted into a Cs_2_SnI_6_ film. The film has a high absorption coefficient (over 10^5^ cm^−1^ from 1.7 eV) and a direct band gap of 1.48 eV, making this an excellent candidate for a lead-free solar cell light absorber. Additionally, this film is air-stable, which further adds to its appeal.

Chen et al. demonstrated the first successful preparation of high-quality Cs_2_TiBr_6_ thin films using a simple vapor-based method (Figure 3d) that can be performed at low temperatures [53]. The thin Cs_2_TiBr_6_ HDP films display various attractive properties, such as a band gap of 1.8 eV, balanced, and extended carrier-diffusion lengths surpassing 100 nm, appropriate energy levels, and significant intrinsic stability in the environment. To form these thin films, a 100 nm thick layer of CsBr was first deposited on the substrate through thermal evaporation, followed by the transfer of the CsBr thin film to a custom-made chamber filled with TiBr_4_ vapor, which was gradually produced by heating TiBr_4_ powder to 200 °C. The entire reaction took 24 h on average to produce phase-pure Cs_2_TiBr_6_. The transformation of the film was driven by solid-state diffusion of Ti^4+^ and Br^-^. Moreover, the film-forming mechanism was dependent on the annealing temperature, with lower temperatures producing incomplete reactions, and higher temperatures causing damage to the CsBr thin film. The study concluded that the optimal annealing condition was 200 °C for 24 h, producing uniform and phase-pure Cs_2_TiBr_6_ HDP thin films with an average grain size of 270 nm and root mean square (RMS) roughness of 24.5 nm. Using this approach, Funabiki and colleagues produced polycrystalline films of (CH_3_NH_3_)_2_SnI_6_ [94]. SnI_4_ and CH_3_NH_3_I were mixed in a 1:2 molar ratio at room temperature using a mortar, where the orange color of SnI_4_ changed to black during mixing with the formation of pure (CH_3_NH_3_)_2_SnI_6_ powder. The powder was then deposited onto silica glass or silicon wafer substrates by evaporation in a vacuum chamber at 120 °C and a pressure of 10^−4^ Torr using a tungsten boat. The cubic (CH_3_NH_3_)_2_SnI_6_ perovskite was an intrinsic n-type semiconductor film with strong visible light absorption. Expanding the lattice with a monovalent cation increased the band gap of the material.

## 4. Vacancy-Ordered HDPs in Solar Cells

### 4.1. As a Transport Layer

**Figure 4:**
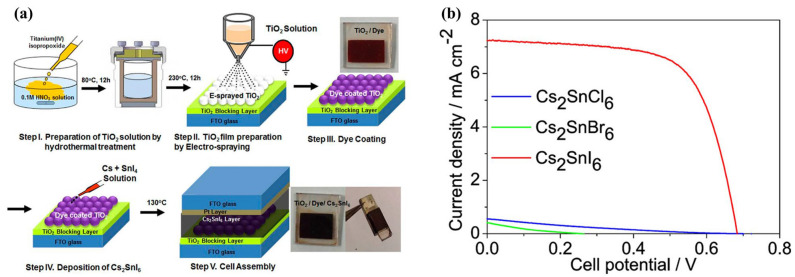
(**a**) Illustration of the fabrication process of DSSC using Cs_2_SnI_6_ as HTM. Reproduced by permission from [51], Copyright 2014 American Chemical Society. (**b**) The J-V curves of Cs_2_SnX_6_-based cells with TiO_2_ (Cl)-TiO_2_ (D/SP) films and the Z907 dye. Reproduced by permission from [95], Copyright 2016 American Chemical Society.

Lee and colleagues first introduced vacancy-ordered HDPs in solar cells, specifically in dye-sensitized solar cells (DSSCs) in 2014 [51]. Cs_2_SnI_6_ was introduced as a promising hole-transporting material (HTM) in DSSCs, demonstrating the potential to replace unstable liquid electrolytes and expensive hole-conducting polymers like spiro-MeOTAD. It showed high efficiency as an HTM and offered a viable alternative to DSSC technology. The molecular salt had a structural similarity to perovskites, such as CsSnI_3_, which also contain Sn^2+^. However, the molecular salt only contained half of its Sn atoms, with the other half missing. This was advantageous because of easy processing in solution and under ambient air conditions. In the experiment shown in Figure 4a, DSSCs were successfully fabricated using Cs_2_SnI_6_ and a mesoporous TiO_2_ sphere film created through an E-spray technique. The use of Cs_2_SnI_6_ significantly reduced the total cell internal resistance, and process optimization obtained an energy conversion efficiency of 4.63% with Z907 dye. This research had significant implications for the field of solar cell technology. Specifically, the study demonstrated that vacancy-ordered HDPs could be effectively used as a material in solar cells. By combining N719 dye with YD2-o-C8 and RLC5 dyes in a more optimized ratio, the efficiency of the cell was boosted to approximately 8% owing to the improved photon confinement. This discovery was noteworthy because it addressed a significant challenge in the development of highly stable PSCs at that time. Kaltzoglou et al. applied mixed-anion vacancy-ordered HDP Cs_2_SnI_3_Br_3_ as an HTM in DSSCs and examined its crystal structure and physicochemical properties [84]. The mixed-halogen Cs_2_SnI_3_Br_3_ had optical properties and hole-transporting efficiency that were more similar to those of Cs_2_SnI_6_ than to those of Cs_2_SnBr_6_. Selecting the correct dye sensitizer and additives was essential for increasing the solar conversion efficiency, which reached a maximum of 3.63% at 1 sun and 7.3% at 0.1 sun using the Z907 dye sensitizer. These perovskites exhibited high efficiency under low solar light irradiation and low hole-transport resistance, making them excellent HTMs A high absorbance of the mixed perovskite across the solar spectrum was observed to contribute to the incident photon-to-current conversion efficiency of the DSSCs. The same group later explored the potential of Cs_2_SnX_6_ perovskites with three different halides (X = Cl, Br, and I) as HTMs in solar cells while investigating their vibrational and optical characteristics [95]. The band gaps of Cs_2_SnCl_6_ and Cs_2_SnBr_6_ had values of 3.9 and 2.7 eV, respectively, while Cs_2_SnI_6_ had a band gap of 1.26 eV. From the J-V curves illustrated in Figure 4b, the devices based on Cs_2_SnCl_6_ and Cs_2_SnBr_6_ materials exhibited poor efficiencies of about 0.07% and 0.04%, respectively. However, the Cs_2_SnI_6_ perovskite demonstrated a significantly higher PCE of 3.30%. The inadequate functioning of cells that use Cs_2_SnCl_6_ and Cs_2_SnBr_6_ can be partially attributed to the formation of sizable crystals from these substances to produce an uneven surface where significant portions of TiO_2_ remain exposed.

**Figure 5:**
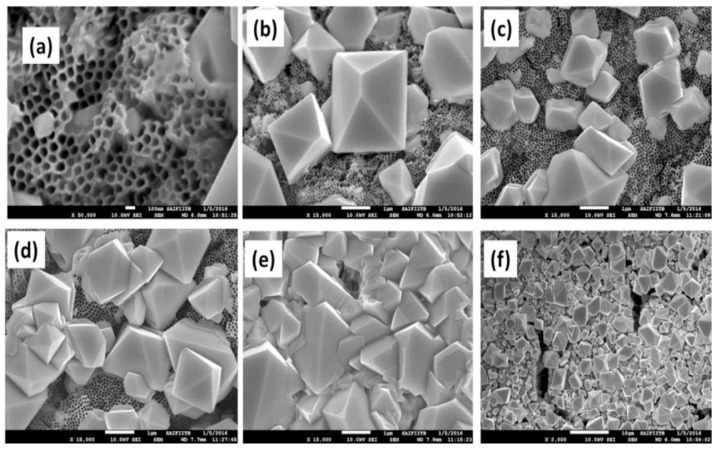
Field emission scanning electron microscopy (FE-SEM) images of Cs_2_SnI_6_ HTM layer deposition on TNT nanotubes for various time intervals, including (**a**) 1 h, (**b**) 2 h, (**c**) 6 h, (**d**) 12 h, and (**e**,**f**) 24 h. Reproduced by permission from [96], Copyright 2018 Elsevier.

Peedikakkandy et al. assessed the synthesis and stability of Cs_2_SnI_6_ and its potential use as an HTM in titania nanotube (TNT)-based DSSCs [96]. The SEM images are shown in Figure 5. The enhanced electron transport properties of vertically aligned TNT-based DSSCs were reported compared with those of TiO_2_ nanoparticulate films, whereas all previous reports had used DSSCs with mesoporous TNT-based photoanodes. This study provided a foundation for exploring and improving the permeability and deposition methods of Cs_2_SnI_6_, a Sn-based air-stable perovskite, as an HTM in TNT-based DSSCs. SnI_2_-doped Cs_2_SnI_6_ HTM was used in the cells to produce an efficiency of 1.33% with 40% fill factor (FF) for a 24 h deposition time while exhibiting improved stability under the ambient air than that of CsSnI_3_ and exhibited thermal stability up to ~80 °C.

Kinoshita et al. used a solution-processed organic–inorganic hybrid film made from (CH_3_NH_3_)_2_SnI_6_ perovskite to develop a wideband solid-state DSSC [97]. Using a MA_2_SnI_6_ perovskite film in solid-state solar cells produced a better sensitivity in the near-infrared range than other HTMs. A comparison of photoelectric conversion characteristics of DSSCs using MA_2_SnI_6_ to those using other HTMs (spiro-OMeTAD, P3HT, and m-MTDATA) revealed that DSSCs with MA_2_SnI_6_ had the highest photocurrent and incident photon-to-electron conversion efficiency, as well as the lowest series resistance, whereas their open-circuit voltage was the lower than that of other HTMs. Additionally, the efficiency of devices using MA_2_SnI_6_ perovskite as the HTM was 0.251%, with an open-circuit voltage (Voc) of 0.242 V, short circuit current density (Jsc) of 2.95 mA cm^−2^, and FF of 0.351. Cs_2_SnI_6_ possessed properties of both n-type and p-type semiconductors depending on the presence of slightly contained Sn^2+^ in the Cs_2_SnI_6_ crystal and the synthesis method employed. MA_2_SnI_6_, synthesized through vacuum vapor deposition, was found to be an n-type semiconductor. However, the conductivity of solution-processed MA_2_SnI_6_ depended on the presence of impurities and synthesis conditions. Changes in the Fermi level of solution-processed MA_2_SnI_6_ were suggested to affect the Voc of DSSCs. Therefore, improving the device performance using MA_2_SnI_6_ perovskite film would rely on the synthesis procedure and doping control.

Khaeroni et al. incorporated Cs_2_SnI_6_ as an electron transport material (ETM) to improve the performance of bulk heterojunction solar cells in the inverted device configuration of ITO/ZnO/Cs_2_SnI_6_/P3HT:PCBM/PEDOT:PSS/Ag. The addition of 2.25 mg/mL of Cs_2_SnI_6_ perovskite as an ETM significantly improved the performance of the solar cell device, with the Jsc increasing from 64.69 to 77.02 mA/cm^2^ and PCE being enhanced from 7.05% to 9.75%. Moreover, the device demonstrated marked stability in the air as evidenced by consistent PCE results four and six weeks after fabrication [98].

### 4.2. As an Active Layer

**Figure 6:**
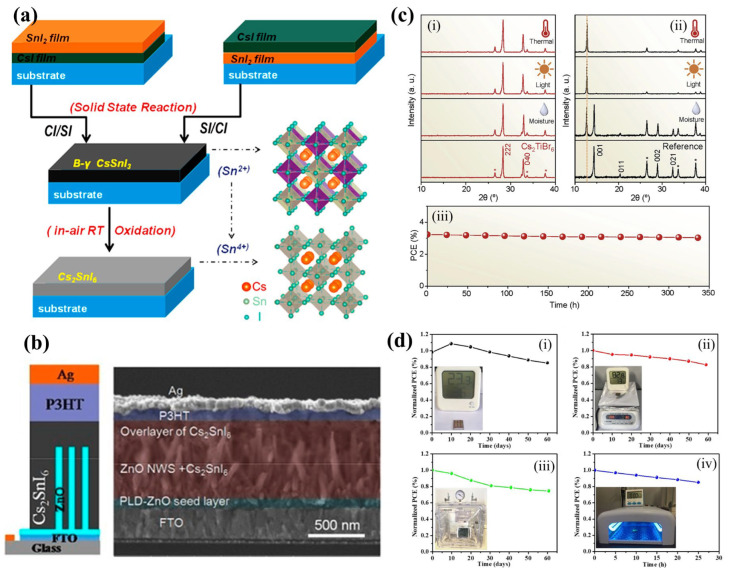
(**a**) The process of growing a Cs_2_SnI_6_ film from CsSnI_3_ using a two-step deposition method based on solid-state reaction with crystal structures. Reproduced by permission from [93], Copyright 2017 Elsevier. (**b**) Structure of FTO/seed layer/ZnO nanorods/Cs_2_SnI_6_/P3HT/Ag solar device and its cross-sectional SEM image. Reproduced by permission from [72], Copyright 2016 John Wiley and Sons. (**c**) X-ray diffraction patterns of (**i**) Cs_2_TiBr_6_ (red) and (**ii**) MAPbI_2_Br (black) thin films before (bottom) and after various stresses, including heat (200 °C, 6 h, and N_2_ atmosphere), light (one-sun, encapsulated), and moisture (23 °C, 80% relative humidity, 6 h) (vertical dashed line indicating the main PbI_2_ peak). The FTO substrate peak is denoted by *. (**iii**) Additionally, PCE of the best Cs_2_TiBr_6_-based PSC (unencapsulated) is plotted against the storage time under environmental stress (70 °C, 30% relative humidity, and ambient light). Reproduced by permission from [53], Copyright 2018 Elsevier. (**d**) Stability test of Cs_2_PtI_6_ under the conditions of (**i**) ambient, 23 °C, 36% RH without encapsulation; (**ii**) high temperature; (**iii**) high humidity; (**iv**) UV radiation. Reproduced by permission from [100], Copyright 2020 American Chemical Society.

In 2016, Cs_2_SnI_6_ was first introduced by Qiu et al. as a light absorber in solar cells. As shown in Figure 6a, unstable B-γ-CsSnI_3_ was oxidatively converted to grow an air-stable Cs_2_SnI_6_ film that was then used as the light absorber in the first planar lead-free perovskite solar cell [93]. The J-V characteristics of devices that used Cs_2_SnI_6_ films were evaluated with different thicknesses. The device with a 300 nm thick Cs_2_SnI_6_ film displayed the highest PCE of 0.96% and photovoltaic parameters of Jsc of 5.41 mAcm^−2^, Voc of 0.51 V, and FF of 0.35. This was the first known demonstration of Cs_2_SnI_6_ as a solar cell absorber material in air, and its performance was superior to that of CsSnI_3_-based planar Schottky cell and was comparable with that of the CsSnI_3_-based mesoporous cell, both of which require a nitrogen glove box for operation. The improved performance was attributed to the optimized thickness and improved film morphology, although the energy-barrier mismatches between the TiO_2_/perovskite/HTM layers may result in inefficient electron/hole extractions, causing low conversion efficiency. Moreover, the very high resistivity of the Cs_2_SnI_6_ films, as determined by unsuccessful Hall measurements, may contribute to the low fill factors of solar cells. The Cs_2_SnI_6_ film was stable upon aging in dry air, but the degradation of solar cell PCE was observed when used as the light absorber. This degradation may be due to material degradation upon applied bias or other materials/interfaces within the device. Organic HTM (e.g., P3HT) has been reported as a problematic source of performance losses. However, the solar cell stability measurements in air showed pronounced stability of the unsealed solar cells for at least one week. This study unveiled novel possibilities for acquiring vacancy-ordered HDP films that are lead-free and that are highly coveted for use in PSCs. The same group later reported the use of Cs_2_SnI_6_ as a light absorber in nanostructure-based PSCs [72]. Figure 6b displays both the device structure and its corresponding cross-sectional SEM image. Pulsed laser deposition (PLD) was the most effective method used for depositing compact ZnO seed layers while spin coating was used for control experiments. The study also showcased the construction of solid-state mesoporous PSCs that employed ZnO nanorod electron transport layers and solution-processed Cs_2_SnI_6_ perovskite films for light absorption. By regulating the precursor concentration, the effect of ZnO nanorod length on device performance was optimized. The study examined how precursor concentrations affected ZnO nanorods and photovoltaic performance using PLD seed layers. Thus, increasing precursor concentration boosted PCE to 0.86% but reduced this to 0.21% when 40 mM was used. The reduction in Jsc was considered to be caused by the quantity of deposited Cs_2_SnI_6_ perovskite, which was attributed to an increase in the pore filling fraction (PFF) resulting from the improved length and pore size of the ZnO nanorod arrays due to an appropriate increase in precursor concentration. This increase in PFF caused a greater amount of perovskite absorber to penetrate the voids and thereby significantly increase the Jsc. Umedov et al. first introduced Cs_2_SnI_6_ absorber in inverted based solar cells [99] and analyzed the effect of substituting Cs^+^ with smaller cations like Rb^+^ and Ag^+^ on the quality of films, performance of devices, and optoelectronic properties of Cs_2_SnI_6_ materials. The study found that incorporating additives in the A-site of the perovskite produced compact and uniform morphology, and the film quality as well as the performance could be improved by optimizing its morphology, defect density, and interface engineering. Consequently, the device that contained Ag^+^ showed the highest PCE of 0.659%, which was significantly greater than those of the pristine and Rb^+^-doped devices (0.103% and 0.258%, respectively).

Chen and colleagues discovered a new aspect in the field of PSCs in 2018 when they conducted an experiment that displayed the capabilities of the Cs_2_TiBr_6_ absorber as a part of the Ti-based vacancy-ordered HDP family [53]. PSCs were created by layering a thin film of Cs_2_TiBr_6_ between TiO_2_ ETM and P3HT HTM using FTO and Au electrodes. The addition of a layer of C60 during the creation of the CsBr thin film enhanced the microstructure by decreasing the grain size and forming a denser grain-boundary network. This improved microstructure facilitated quicker ion diffusion, producing a high-quality Cs_2_TiBr_6_ HDP thin film with reduced RMS roughness of 14.6 nm. The study implied that manipulating the film microstructure is an effective method for enhancing Cs_2_TiBr_6_-based PSCs. In addition, the highest achieved PCE of 3.3% was reported, marking the best result ever reported for vacancy-ordered HDP solar cells. The stability of Cs_2_TiBr_6_ HDP thin films, both intrinsically and environmentally, was discussed in comparison with other halide perovskites such as MAPbI_3_, MAPbBr_3_, and MASnI_3_. An X-ray diffraction study revealed that Cs_2_TiBr_6_ HDP thin films exhibited superior tolerance to heat, moisture, and light while sustaining phase purity for a more extended period compared with halide perovskites (Figure 6c). This stability was linked to the all-inorganic nature of Cs_2_TiBr_6_ and the stability of the titanium cation in the preferred +4 oxidation state. Figure 6c demonstrates that an unencapsulated PSC device retained 94% of its initial PCE and maintained a PCE of 3.03% after 14 days of storage. They also evaluated the stability of the PSC device was assessed by subjecting this to continuous one-sun illumination, which showed an 85% retention of PCE. Consequently, the Cs_2_TiBr_6_ HDP thin films were suggested as promising candidates for PSCs in tandem PV applications, owing to exceptional intrinsic and environmental stability.

Yang and colleagues suggested a novel solar cell configuration that employed a Cs_2_PtI_6_ film of high quality and a narrow band gap [100]. A high-quality Cs_2_PtI_6_ film with desirable properties, such as a high absorption coefficient, broad absorption range, and an optical band gap of 1.37 eV, was used in a solar cell with a conventional planar heterojunction structure of ITO/SnO_2_/Cs_2_PtI_6_/Spiro-OMeTAD/Au. To ensure a smooth and uniform film without pinholes, hydroiodic acid was added to the precursor solution during film fabrication. The solar cell without HI had a Jsc of 1.2 mA/cm^2^, a Voc of 0.61 V, an FF of 0.48, and a PCE of 0.35%. However, after optimization, the Voc, Jsc, and FF values improved to 0.73 V, 1.2 mA/cm^2^, and 0.82, respectively, producing a higher PCE of 0.72%. Despite the lower Jsc due to the indirect band gap and poor film quality of the material, significant enhancements were observed in Voc and FF. Stability tests showed that the Cs_2_PtI_6_-based solar cells maintained approximately 80% of their initial efficiency even after 60 days under various conditions, including ambient conditions, high temperature, high humidity, immersion in water, and exposure to ultraviolet radiation (Figure 6d). The stability of these Cs_2_PtI_6-_based solar cells was superior to other known halide PSCs during the same period of study.

## 5. Simulation Studies

**Figure 7:**
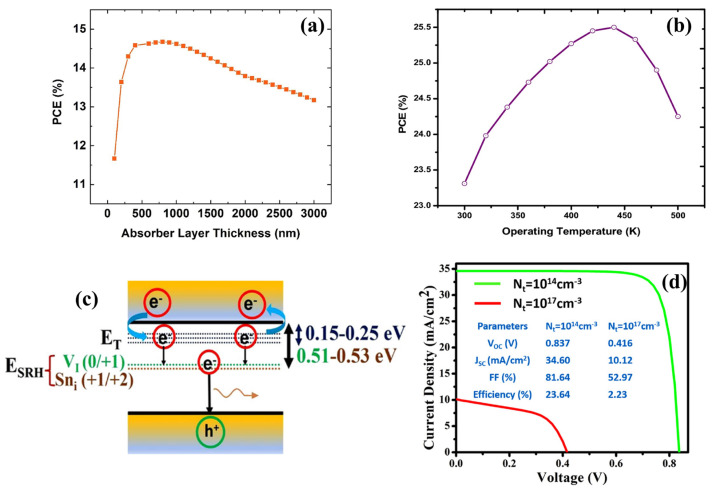
PCE of the simulated (**a**) Cs_2_TiBr_6_-based device with varying absorber thickness (Reproduced by permission from [102], Copyright 2020 Elsevier) and (**b**) Cs_2_SnI_6_-based device with operating temperature. Reproduced by permission from [104], Copyright 2022 Springer Nature. (**c**) Illustration depicting notable flaws within the Cs_2_SnI_6_ double perovskite are presented, along with the confinement of carriers in different defect states. (**d**) J-V curves of Cs_2_SnI_6_-based devices with various defect densities. Reproduced by permission from [105], Copyright 2022 John Wiley and Sons.

Samanta et al. performed the initial simulation of Cs_2_TiBr_6_ PSC in 2020 by using the Silvaco ATLAS device simulator [101]. The study investigated the influence of different thicknesses and doping levels of electron and hole-transport layers on the performance of Cs_2_TiBr_6_-based solar cells. The simulation showed that NiO was the most efficient HTM, and doping of transport layers could significantly enhance the device’s performance. The thickness of TiO_2_ ETM was chosen as 10 nm based on its role as a window layer, whereas the thickness of NiO HTM was chosen as 10 nm to avoid cracks and achieve the highest fill factor. The study also examined the effects of different anode materials on device performance, showing that Au performed better than Ag and Cu. However, the simulated results were below the theoretical limit, which was possibly due to the presence of glass substrates and defects in real solar cell structures. A Cs_2_TiBr_6_-based PSC that used doped TiO_2_ (10 nm) and NiO (10 nm) layers produced a PCE of 5.5% as opposed to the only available report on Cs_2_TiBr_6_-based PSCs on that time which demonstrated a PCE of 3.3%. The final structure that demonstrated the most effective performed was FTO/n-TiO_2_ (10 nm)/Cs_2_TiBr_6_ (330 nm)/p-NiO (10 nm)/Au, which yielded a PCE of 8.5%. Jani et al. conducted simulation studies in the same year to investigate the feasibility of using Cs_2_TiBr_6_ as the active layer and Cu_2_O as the HTM [102]. The wx Analysis of Microelectronic and Photonic Structures (wxAMPS) software was employed for the numerical analysis. The study examined the relationship between the PCE and the absorber thickness within 300–3000 nm (Figure 7a) and determined that an optimal perovskite layer thickness of 800 nm produced a maximum PCE of 14.68%. The study also revealed that increasing the absorber layer thickness decreased the saturation current and increased the Voc. The results suggested that compositional changes in the perovskite through doping methods could improve the PCE by up to 20.49%. Similarly, Ahmad and colleagues used CdTe as a HTM in their simulation investigating Cs_2_TiI_6-_based solar cells [103]. Calculations were performed using the solar capacitance simulator (SCAPS-1D). A new perovskite solar cell design (Au/p-CdTe/Cs_2_TiI_6_/n-TiO_2_/ITO) was proposed and then optimized through simulation. The power conversion efficiency was 15.06%, and parameters such as mobility, defect density, and energy band gap were reported. The research emphasized the importance of sustainable energy research, particularly in multilayer inorganic halide solar cells.

Chauhan et al. introduced and demonstrated a new device structure, FTO/GO/Cs_2_SnI_6_/Cu_2_O/Au, that exhibited remarkable performance [104]. The effects of several factors such as absorber thickness, transport layers thickness, defect density, and working temperature, and confirmed that these factors impacted the efficiency of the solar cells were carefully examined. Figure 7b displays the PCEs obtained by altering the operating temperature of a simulated PSC in 11 increments of 20 K from 300 to 500 K. The PCE and Voc increased from 23.31% to 25.33% and from 1.25 to 1.27 V, respectively, until the temperature reached 460 K. However, further temperature increases reduced the PCE performance. The photovoltaic performance improved due to the increased thermal energy, which resulted in a higher number of carriers. However, the decrease in performance occurred as a result of increased recombination at the interface, impacting the band gap, carrier mobility, and carrier concentrations. The effect of series resistance on the photovoltaic performance of two different configurations of Cs_2_SnI_6_-based solar cells was also investigated. The series resistance was varied from 10 to 100 ohm.cm^2^ in ten steps and observed a significant impact on the performance of the device, including the current-voltage curve, FF, and PCE. The experimental configuration exhibited a low PCE due to by the large series resistance, which indicated that performance could be improved by reducing the series resistance.

Similarly, Porwal et al. investigated Cs_2_SnI_6_ double perovskite-based photovoltaic devices with various HTMs, including NiO, CuO, Cu_2_O, CuI, CuSCN, CuSbS_2_, PEDOT: PSS, PTAA, spiro-OMeTAD, and graphene oxide, as the ETM. The Cu_2_O HTM-based device exhibited the highest PCE of 23.64%, with a Voc of 0.837 V, Jsc of 34.6 mA cm^−2^, and FF of 81.64% [105]. The role of defects density and other factors such as doping density, light intensity, ETM thickness, operating temperature, and parasitic resistances were also investigated. The simulation also investigated the impact of defects on the performance of solar cells made from halide perovskites. Defects created energy states near the valence or conduction band edges to affect carrier lifetime and mobility, which caused recombination. Gaussian distribution equations were used to determine the energy levels and densities of these defects. The Shockley–Read–Hall (SRH) recombination model was employed to illustrate the importance of defect energy states and their concentration for device performance (Figure 7c,d). Deep energy level defects were primarily responsible for SRH nonradiative recombination and reducing minority carrier lifetime.

**Figure 8:**
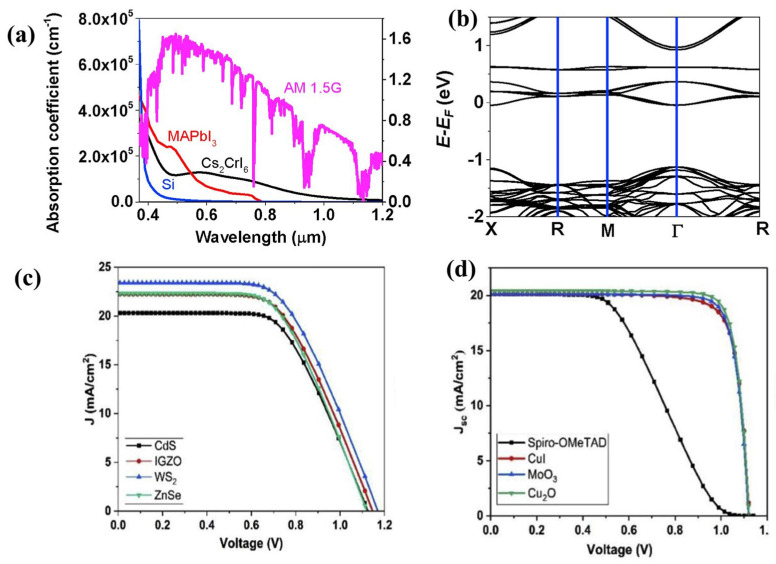
(**a**) The absorption coefficient of Cs_2_CrI_6_, MAPbI_3_, and Si compared in relation to the solar spectrum (**b**) Band structure of Cs_2_CrI_6_ calculated using Heyd–Scuseria–Ernzerhof density functional calculations. Reproduced by permission from [106], Copyright 2021 Elsevier. J-V measurement for Cs_2_PtI_6_-based solar cells assembled with (**c**) different HTMs and (**d**) different ETMs. Reproduced by permission from [107], Copyright 2022 Elsevier.

Zhao et al. proposed a new type of vacancy-ordered HDP Cs_2_CrI_6_ to achieve space solar cells with superior performance [106]. This study compared the photovoltaic performance of Cs_2_CrI_6_ and MAPbI_3_ perovskite solar cells. Cs_2_CrI_6_ had a higher absorption coefficient and wider absorption cutoff than MAPbI_3_ (Figure 8a), producing greater photon capture and a higher Jsc. The Voc decreased with increasing perovskite thickness owing to the decline of electron and hole quasi-Fermi levels, whereas the FF remained consistent. The band structure in Figure 8b obtained using the Heyd–Scuseria–Ernzerhof density functional calculations reveals that Cs_2_CrI_6_ possesses a direct band gap. Cs_2_CrI_6_ was also suitable as the bottom cell in monolithic all-perovskite tandem solar cells because of its narrow band gap (1.08 eV), and the highest device performance was achieved with a tandem structure of FTO/ poly(3,4-ethylenedioxythiophene) polystyrene sulfonate (PEDOT: PSS)/MAPbI_3_/C60/ITO/PEDOT: PSS/Cs_2_CrI_6_/C60/Ag. The material exhibited excellent optical absorption, high carrier mobility (~10^3^ cm^2^/V), and low capture cross-section, which produced ultrahigh PCEs for single-junction (22.4%) and monolithic all-perovskite tandem solar cells (26.6%). The findings also indicated that Cs_2_CrI_6_ could endure proton irradiation of up to 10^16^ p.cm^−2^, implying that it had potential as a viable option for use in photovoltaic cells and their space-related applications.

AbdElAziz et al. conducted simulation studies on the platinum-based vacancy-ordered HDP Cs_2_PtI_6−x_Br_x_ (x = 0, 2, 4, and 6) to examine the performance of solar cells that use different types of HTM and ETM [107]. Cs_2_PtI_6_ material was suitable for single-junction solar cells in PV applications while other Br-doping materials were suitable for use as a top cell in tandem solar cells with appropriate engineering. To model an all-inorganic Cs_2_PtI_6_-based solar cell, different inorganic materials for the HTM and ETM were simulated using the SCAPS-1D simulator. Suitable HTMs and ETMs were selected based on the favorable band alignment matching with the lowest unoccupied molecular orbital (LUMO) and highest occupied molecular orbital (HOMO) band edges of the Cs_2_PtI_6_ material. The Cu_2_O HTM performed better than other HTMs, and WS_2_ was identified on the basis of the simulation results as the most promising ETM for the Cs_2_PtI_6_ solar cell (Figure 8c,d. Further optimization of the device was conducted by testing different metalwork functions, which determined that the device performed best with metalwork functions higher than 4.9 eV. Additionally, the device was optimized for Cs_2_PtI_6_ to demonstrate that a 400 nm absorber layer thickness and 10^20^ cm^−3^-doping concentration yielded the best performance. The device also exhibited the most effective performance with a defect density concentration of 1 × 10^12^ cm^−3^. Thus, the best achievable efficiency of 17.2% for an FTO/WS_2_/Cs_2_PtI_6_ (400 nm)/Cu_2_O/carbon solar cell was identified.

## 6. Conclusions and Outlook

This review provides an overview of the latest advancements and potential strategies in substituting lead-based perovskites with vacancy-ordered HDP materials in solar cell applications. We focused on discerning the impetus or driving force behind the progress that has been made concerning these compounds thus far. The review provided a comprehensive analysis of various synthetic approaches for materials and methods of film preparation and their impacts on material properties and device performance. Detailed information on recent simulation studies is also provided, offering valuable insights for the experimental development of high-performance vacancy-ordered HDP-based solar cells.

From a literature review perspective, lead-free vacancy-ordered HDPs clearly have both advantages and disadvantages. Currently, this type of compound has only had a positive impact on the toxicity and ambient stability of perovskite solar cells. Even though these materials have already achieved noteworthy nontoxicity and ambient stability, their photophysical properties and PV performance still fall short of expectations. To fully exploit the potential of these materials and use them as the best possible alternative to lead-based perovskites, new discoveries are required regarding their structural features, morphology control, fundamental photo-physics, tuning of optoelectronic properties, preferred orientation, and device optimization.

Theoretical computations revealed that specific vacancy-ordered and lead-free HDP materials possess a promising potential for outstanding PV properties. Therefore, further research combining theoretical and experimental approaches is crucial to advance their practical applications. Currently, no effective strategies have been implemented to improve the performance of vacancy-ordered HDP solar cells, and the only approach so far used is to replace the absorber layer of the solar cell with various types of vacancy-ordered HDP materials and adjust the stoichiometry of the layer. To achieve the desired outcome, several avenues of investigation must be proposed and evaluated. These include exploring different methods of vapor deposition to produce thin films of superior quality, converting indirect band gap configurations to direct ones through doping, adjusting band gaps by incorporating halogens and pnictogens with larger atomic radii into the X and B sites, ensuring high electronic dimensionality, and identifying optimal crystal growth conditions to minimize the occurrence of point defects. The development of a halide perovskite substance that integrates the exceptional light-capturing capabilities of lead-based perovskites with the stable and nonhazardous features of HDPs would represent a significant advancement in the domain of solar cells.

## Data Availability

In this review manuscript, no new data were created.
